# With Appreciation

**DOI:** 10.1002/jcsm.12664

**Published:** 2020-12-19

We thank the following individuals who served as manuscript reviewers for The Journal of Cachexia, Sarcopenia and Muscle (JCSM) in 2020.

We highly appreciate their efforts. Fair, conscientious and timely peer reviews contribute to the success of JCSM.

The Editors.

### 6 and more reviews

Matthew Alexander

Stephen Alway

Andrew Coats

Paola Costelli

Craig Goodman

Hidetaka Wakabayashi

Tobias Wollersheim

### 3–5 reviews

Takashi Abe

Volker Adams

Stefan Anker

Vickie E Baracos

Mauricio Berriel Diaz

Bert Blaauw

Andrea Bonetto

T. Scott Bowen

Jeffrey Brault

James Carson

Robson Carvalho

Arrigo Francesco Giuseppe Cicero

Paul Coen

Eduardo Costa

Jackie Dawson

Wolfram Doehner

Rosario Donato

H. Alexander Ebhardt

Sven Geissler

Marcus Goncalves

Antoneta Granic

Nicholas Greene

Denis Guttridge

David Hammers

Michiyoshi Hatanaka

Joshua Huot

Satoshi Ikeda

Andrew R. Judge

Toshimi Kaido

Seyyed Mohammad Reza Kazemi‐Bajestani

Andy Khamoui

Markus Luedi

Frank Misselwitz

Naoharu Mori

Anselmo Moriscot

Kevin Murach

Anna Pakula

Fabio Penna

Marcelo Pereira

Dominik Pesta

Emidio Pistilli

Kunihiro Sakuma

K Sakuma

Joerg Schefold

Marília Seelaender

Pietro Spitali

Jochen Springer

Peter Szentesi

Sara Tagliaferri

Erin Talbert

Francesco Saverio Tedesco

Brennan Thompson

David van Dijk

Maurizio Volterrani

David Waning

Yefei Wen

Tobias Winkler

Chih‐Hsing Wu

### 1–2 reviews

Eeva Aartolahti

Santanasto Adam

Odessa Addison

Daniel Aeberli

Yoshihiro Akashi

Anjalee Thanuja Amarasekera

Markus Anker

Yoshitsugu Aoki

Hidenori Arai

Anne‐Sophie Armand

Atsushi Asakura

Didier Attaix

Hideo Baba

Martin Bahls

Christine Baldwin

Rocco Barazzoni

Thiago Barbosa‐Silva

Gil Bar‐Sela

Elisabeth Barton

Juergen Bauer

Jeannette Beasley

Charlotte Beaudart

Paul Beckers

Libera Berghella

Pengpeng Bi

Lara Bianhi

Gianni Biolo

Roméo Blanc

Jose‐Ramon Blanco

Simina Boca

Richard Bohannon

Danilo Bondi

Elisabet Borsheim

Federico Bozzetti

Scott Brakenridge

Leigh Breen

Uli Broedl

Marco Brotto

Egon Burian

Gillian Butler‐Browne

Eugenia Carvalho

Peggy Cawthon

David Cella

Joe Chakkalakal

Hsin‐Hung Chen

Andrew Clark

Peter Claus

Enrico Clini

Dario Coletti

Vsile Coman

Miranda Cullins

Olof Dahlqvist Leinhard

Radbod Darabi

Srinivasan Dasarathy

M. Davis

Gangarao Davuluri

Katrien De Bock

Boel De Paepe

Marian de van der Schueren

Hans Degens

Louise Deldicque

Elise Deluche

Bijan Dey

Christine DiDonato

Francesco Dioguardi

Marlou Dirks

Sandor Dorgo

Hervé Dubouchaud

Stephanie Duguez

Jean‐Francois Dumas

Esther Dupont‐Versteegden

Jean El‐Cheikh

Nir Eynon

Alessandro Fanzani

Julio Ferreira

Stephan Fichtlscherer

Nicoletta Filigheddu

Merran Findlay

Florian Fintelmann

Dirk Fischer

Sue Fletcher

James Fluckey

Brian Focht

Martino V. Franchi

Gabriel Francisco Pereira Aleixo

Paula Freire

Armin Frille

Nicholas Fuggle

Arata Fukushima

Davide Gabellini

Imed Gallouzi

Jose Garcia

Giacomo Garibotto

Corinna Geisler

Loncar Goran

Brad Gordon

Gilles Gouspillou

Neil Greening

Mark Griffiths

Ewout Groen

Michael Grusch

Jenny Gunton

Cheng Guo

Jack Guralnik

Dirk Habedank

Jana Haberlova

Mark Hamrick

Jin Han

Mohamed Hankir

Justin Hardee

Nicolas Hart.

Shinji Hatakeyama

Klaus Hauer

Daniel Hirai

Eric Hoffman

David Hood

Nicholas Hopkinson

Charles Hoppel

Keisuke Horiuchi

Ping Hu

Megan Huisingh‐Scheetz

Juha Hulmi

Chikwendu Ibebunjo

Hikaru Ihira

Alp Ikizler

Rodney Infante

Marco Invernizzi

Mikel Izquierdo

Connie Jackaman

Carsten Jacobi

Anita Jankowska

Aminah Jatoi

Cathleen John

Boris Jung

Friederike Jungmann

Stephen Kaplan

Takao Kato

Anu Kauppinen

Takumi Kawaguchi

Paul Kemp

Justin Keogh

Grahame Kidd

Douglas Kiel

Won Kim

Hiroshi Kimura

Nicole Kiss

Fumitaka Koga

Stephen Kolb

Masaaki Konishi

Alexander Krannich

Shihuan Kuang

Siegfried Labeit

Maciej Lalowski

Francesco Landi

Brittney Lange‐Maia

Lars Larsson

Michaël Laurent

Fulvio Lauretani

Alessandro Laviano

Carl Lavie

Jie Lee

Alessia Lena

Paul Lewandowski

Chao‐Hsiung Lin

Thomas Lisse

Ping‐Yen Liu

Hanns Lochmüller

Antonio Macciò

Juliano Machado

Abigail Mackey

Keisuke Maeda

Robert Mak

Giovanni Mantovani

Daniela Mari

Daniel Marks

Megan Marron

Lisa Martin

Anna Maria Martone

Zoltan Mathe

Giulia Maurizi

Merry‐Lynn McDonald

William E. Mitch

Junaith Mohamed

Alex Molassiotis

Pablo Molina

Daniel Moore

Hamid Moradi

Beatrice Morio

John Morley

Vincenzo Musolino

Gustavo Nader

Ashok Narasimhan

Marco Narici

Francisco Naya

Enzo Nisoli

Dominik Nörenberg

Kristina Norman

Yoshitsugu Obi

Julien Ochala

Thomas OConnell

Kay Ohlendieck

Steven Olde Damink

Patrick Owen

Daniela Palacios

Adelaida Palla

Frédéric Pamoukdjian

Lynn Panton

Antonio Paoli

Michael Paris

Jongha Park

Evasio Pasini

Bhoomika Patel

Tao‐Chun Peng

Peter Penson

Goncalo Pereira

Rosanna Piccirillo

Gustavo Pimentel

Michael Polkey

Karteek Popuri

Simone Porcelli

Keith G Professor

Olga Prokopchuk

Sarah Purcell

Zudin Puthucheary

Joe Quadrilatero

Jens Reimann

Sander Rensen

Francesca Riuzzi

Vanina Romanello

Sabrina Ronen

Giuseppe Rosano

Hamilton Roschel

Shannon Rose

Eric Rullman

Maurilio Sampaolesi

Srihari Sampath

Kiyoshi Sanada

Marco Sandri

Nadja Scherbakov

Cameron Schmidt

Stylianos Scordilis

David Scott

Robert Seaborne

Jochen Seitz

Adam Sharples

Gary Sieck

Mirko Signorelli

Maria Ines Silva

Rajan Singh

Richard Skipworth

T. Smith

Athanassia Sotiropoulos

Guro Stene

Florian Strasser

Florian Streitparth

Daniel Taillandier

Tetsuya Takamasu

Nicolas Tardif

Marine Theret

Jeanette Thom

Martine Thomis

Matteo Tosato

Michael Toth

Jose Trevino

Trupti Trivedi

Jorn Trommelen

Emmanuel Tsochatzis

Kohjiro Ueki

Miroslava Valentova

Ans T van der Ploeg

Ardy van Helvoort

Tom Vanden Berghe

José M. Villalba

D.C. Vodnar

Stefano Volpato

Henning Wackerhage

Glenn Walter

Xiaonan Wang

Christopher Ward Ward

Kate Ward

Steven Welc

James White

Kenneth Wilund

Simon Wing

Carol Witczak

Miles Witham

Stefan Wohlfeil

Ricardo Xavier

Hong Xia Xu

Qian‐Li Xue

Sumio Yamada

Mee‐Sup Yoon

N.E. Zanchi

Werner Z'Graggen

Qi Zhao

Yao Zhu

Cheng‐le Zhuang

Jana Zschüntzsch

芷萍 鍾

